# Diet Quality of Adolescents and Adults Who Completed the Australian Healthy Eating Quiz: An Analysis of Data over Six Years (2016–2022)

**DOI:** 10.3390/nu14194072

**Published:** 2022-09-30

**Authors:** Megan Whatnall, Erin D. Clarke, Marc T. P. Adam, Lee M. Ashton, Tracy Burrows, Melinda Hutchesson, Clare E. Collins

**Affiliations:** 1 School of Health Sciences, College of Health, Medicine and Wellbeing, University of Newcastle, Callaghan, Newcastle, NSW 2308, Australia; 2 Food and Nutrition Research Program, Hunter Medical Research Institute, New Lambton Heights, Newcastle, NSW 2305, Australia; 3 School of Information and Physical Sciences, College of Engineering, Science and Environment, University of Newcastle, Callaghan, Newcastle, NSW 2308, Australia; 4 School of Education, College of Human and Social Futures, University of Newcastle, Callaghan, Newcastle, NSW 2308, Australia; 5 Active Living Research Program, Hunter Medical Research Institute, New Lambton Heights, Newcastle, NSW 2305, Australia

**Keywords:** diet quality, online, dietary assessment

## Abstract

Diet quality is influenced by demographics and can change over time. This study aimed to (1) compare diet quality among adolescents/adults who completed the online Healthy Eating Quiz (HEQ) by demographic characteristics, and (2) to evaluate change in score over time for repeat completers. HEQ data collected between July 2016 and May 2022 were analysed, including demographics (age, gender, vegetarian status, socio-economic status, number of people main meals are shared with, country), and diet quality calculated using the Australian Recommended Food Score (ARFS) (range 0–73) for respondents aged ≥ 16 years. Differences in ARFS by demographic characteristics and change in score over time, adjusted for age, gender and vegetarian status, were tested by linear regression. The participants (*n* = 176,075) were predominantly female (70.4%), Australian (62.8%), and aged 18–24 years (27.7%), with 4.0% (*n* = 7087) repeat completers. Mean ± SD ARFS was 33.9 ± 9.4/73. Results indicate that ARFS was significantly lower among males and significantly higher with increasing age group, higher socio-economic status, in vegetarians, those who shared main meals with others, and those living in Australia (*p*-values < 0.001). Mean change in ARFS over time (2.3 ± 6.9) was significantly higher for those with lower baseline scores (*p* < 0.001). Publicly available, brief dietary assessment tools have the potential to improve diet quality at the population level.

## 1. Introduction

Diet quality is a term used to describe the variety of foods usually consumed across food groups that are aligned with national dietary guidelines [1]. Diet quality can be assessed using an a priori diet scoring tool or index or post hoc using a data-driven analysis to identify dietary intake patterns [1,2]. Diet quality scores can be based on food and/or nutrient intakes, with higher diet quality scores aligning with a healthier dietary pattern that includes a greater variety and intake of nutrient-dense foods such as fruit, vegetables, wholegrains and sources of lean protein [1]. Poor diet quality including inadequate intakes of nutrient-dense foods and excessive energy-dense, nutrient-poor foods such as ultra-processed foods, is associated with an increased risk of cardiovascular disease (CVD), type 2 diabetes, and some cancers [3,4], as well as increased healthcare claims and costs [5,6]. For example, systematic reviews have identified higher diet quality to be associated with 14–29% reductions in CVD risk [1,7]. Diet quality in many countries is not optimal with a large percentage of the population not meeting national recommendations for fruit, vegetables and wholegrains and consuming energy-dense, nutrient-poor foods in excess [8,9]. In Australia for example, adults consume approximately one-third of daily energy intake from nutrient poor foods [10].

Diet quality is influenced by individual food choices and demographics, such as gender, vegetarian status, socioeconomic status and the social context of eating [11,12,13]. Females compared with males are more likely to have a healthier diet [12,14,15,16]. This may be due to greater interest in health among females, and the influence of this on the healthfulness of individual food choices. People following a vegetarian diet, whether for ethical or health reasons, can have a healthier dietary intake due to higher intakes of plant-based sources of protein, fruit, vegetables and wholegrains in place of animal-based foods [17], although this is not always the case [18]. Those of higher socioeconomic status are more likely to have a healthier diet compared to those of lower socioeconomic status [16,19,20,21]. This relationship is mediated partly by lower levels of food literacy among those with lower diet quality, and barriers with access, affordability and time available to purchase, prepare and consume healthier foods, often leading to less healthy food choices [12,21,22]. The number of people with whom meals are shared can also influence dietary pattern. Those consuming meals alone are more likely to have a less varied diet, and therefore a lower diet quality, than those who share meals with others where cooking for a number of people can be more efficient and economical [23,24,25].

Food choices and diet quality, as well as the factors which influence them, can also change over time [26] and with intervention [27], such as behaviour change interventions. For example, food choices often change as individuals transition through different life stages, with factors such as living and financial situations, social relationships and health priorities changing [26]. These factors then influence the types and variety of foods consumed, and therefore diet quality. Further, behaviour change interventions as minimal as a single session have shown to trigger positive short-term changes in dietary behaviours and diet quality in adults including increased fruit and vegetable and decreased total fat intakes [27]. Regardless of intervention duration or intensity, the evidence demonstrates that more effective behaviour change interventions are those that are tailored and personalised and include active and practical components such as goal setting, feedback mechanisms, and planning and practising skills and behaviours [27,28,29]. Further, Thomson et al. suggest that achieving and sustaining dietary behaviour change may be more effective where interventions include elements such as social marketing, and utilise technology [30]. Consideration of change in dietary intakes and quality over time is a critical aspect of understanding the factors which influence them, and how to positively change individual and population dietary intakes.

The aim of the current paper is to compare diet quality among adolescents and adults who have completed the online, publicly available, diet quality index—the Healthy Eating Quiz (HEQ)—by demographic characteristics (age, gender, vegetarian status, socio-economic status, number of people main meals are shared with, country of residence). A second aim is to evaluate the change in diet quality score over time for those who return to repeat the HEQ. The HEQ is a brief diet quality index tool which assesses dietary variety across food groups aligned with the Australian Dietary Guidelines [31], and provides automated and tailored dietary feedback and advice.

## 2. Materials and Methods

### 2.1. Study Design and Participants

**Figure 1:**
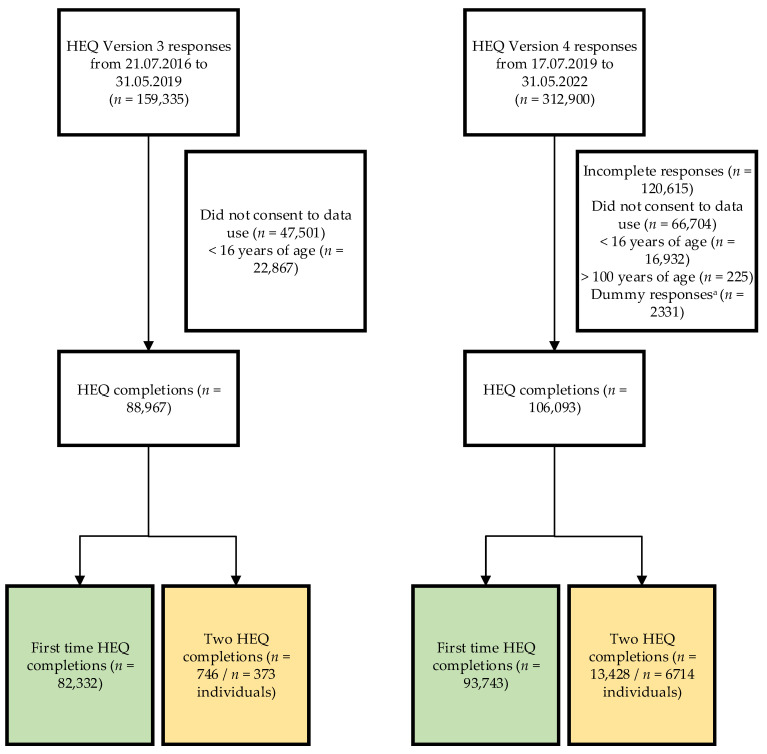
Flow diagram of participants. ^a^ Dummy responses include test completions by the research team, non-authenticated email addresses.

This study analysed data from an online diet quality index tool: the HEQ (www.healthyeatingquiz.com.au, accessed on 30 September 2022). The HEQ is described in detail in the materials section below. The study design is cohort with reporting of cross-sectional and longitudinal data. Those who completed the HEQ once are included in the cross-sectional analysis, and those who returned to the website and repeated the HEQ at least three weeks after their first completion are also included in the longitudinal sub-group analysis. The online HEQ has been refined over time and this paper reports on those who completed version three of the HEQ (available online from 2016–2019) and the current version four (available online from 2019-current). There were no changes to the scoring between versions. Study participants include those who completed the HEQ between 21 July 2016 and 31 May 2022. Inclusion criteria were: those who completed the HEQ, gave consent for their data to be used for research purposes and were aged 16 years or older. Respondents were excluded if they had incomplete data, did not consent to data use, or were aged < 16 years. For version four only, respondents were also excluded if they were aged > 100 years (*n* = 225) due to small numbers and as it was deemed implausible that people of this age would complete the HEQ. This was able to be determined in version four as age is collected as a number, where in version three age group was collected as described in the materials section below. A detailed breakdown of participant numbers for the cross-sectional and longitudinal analyses, with reference to inclusion criteria, is provided in Figure 1. The HEQ is freely available online and regularly promoted by the research team, for example via online media, radio and within Massive Online Open Courses (MOOCs) and research studies. Recruitment of individuals to complete the HEQ is therefore organic and occurs through various strategies and platforms. These various recruitment strategies have been used across all versions of the HEQ. Respondents are encouraged to return and complete the HEQ by email bi-monthly after first completing, and therefore this is an additional recruitment strategy relevant to those included in the longitudinal sub-group analysis. Ethics approval for the study was obtained from the University of Newcastle Human Research Ethics Committee (H-2016-0168). The conduct and reporting of this work complies with STROBE-nut guidelines [32] (Table S1).

### 2.2. Materials

#### Healthy Eating Quiz

The HEQ includes 70 questions and assesses dietary variety in line with each of the food groups in the Australian Dietary Guidelines [31]. The HEQ takes approximately seven minutes to complete and provides automated scoring and feedback on ways to improve diet quality. The HEQ was modelled on the Australian Recommended Food Score (ARFS) [31], and is comprised of eight sub-scales and a total score from 0–73. The sub-scales and respective maximum scores are vegetables (21 points), fruit (12 points), meat/flesh foods (7 points), plant-based protein foods (6 points), breads and cereals (13 points), dairy foods (11 points), water (1 point), and spreads and sauces (2 points). The HEQ is calculated by summing the points for each item, with most items scored one point for consumption frequency of ≥once per week. Scoring is adjusted for individuals who report they follow a vegetarian diet, with a score of zero for the meat/flesh food items and doubled points for the plant-based protein food items as well as bonus points for consuming soybeans or tofu and other beans or lentils at least once per week or more. A higher score is indicative of greater diet variety, and scoring feedback is also categorised as <33, 33–38, 39–46, or ≥47. The brief, automated dietary feedback includes total and sub-scale scores, tailored score comparisons with others of the same age and gender, and practical advice on how to increase diet variety and quality across each sub-scale (e.g., to improve breads and cereals score; ‘Serve brown rice or wholemeal noodles with a stir fry for a change’, and to improve plant-based proteins score; ‘Start by replacing half the meat in a stir-fry or casserole or bolognese sauce with canned or dried beans, lentils and chickpeas’). Further detail on the HEQ and validation studies of the ARFS have been previously published [31,33,34].

### 2.3. Demographics

Demographic characteristics captured in the HEQ include age, gender, postcode of residence, country of residence, whether respondents follow a vegetarian diet (yes/no), and how many people they share their main meals with (themselves; one other person; two or more other people). The following questions differed slightly between versions three and four of the HEQ. Age was collected as age groups in version three and month and year of birth in version four. For consistency age is reported as age groups in this paper (16–17 years; 18–24 years; 25–34 years; 35–44 years; 45–54 years; 55–64 years; 65–74 years; ≥75 years). Gender was collected as male or female in version three, with an additional response option of ‘another gender identity’ added in version four. Country of residence and main meal sharing questions were optional to complete in version three but required a response in version four. Postcode data are used only for respondents in Australia as a measure of socioeconomic status, and this was optional in both versions but prompted for those in Australia in version four. Postcode is matched to the Australian Bureau of Statistics, Index of Relative Socioeconomic Advantage and Disadvantage (IRSAD) data [35]. IRSAD is a summary index which considers the economic and social conditions of individuals and households within a geographical area. IRSAD ranges from 1–10, with one indicating the most disadvantage and 10 the most advantage in terms of economic and social conditions.

### 2.4. Statistical Analysis

Statistical analyses were conducted using Stata software version 14.2 (StataCorp, College Station, TX, USA). Descriptive statistics are reported as number and percentage or mean and standard deviation (SD). Mean (SD) scores are reported for ARFS total and sub-scale scores, with the exception of the water and spreads and sauces sub-scales as the score ranges for these are narrow (0–1 and 0–2, respectively), and mean (SD) values are less insightful. Differences in total ARFS by demographic characteristics was tested by linear regression. Repeat completion of the HEQ was defined as those who completed the quiz twice at least three weeks apart, and these data were paired for further analysis. Change in total and sub-scale scores were calculated as score at second completion minus score at baseline for each repeat completer. For those who completed the quiz more than twice (*n* = 2080), only the first and next completion that was at least three weeks later were included for the purposes of this analysis. Change in total ARFS by score category at baseline was tested by linear regression, including an unadjusted model and a model adjusted for age, gender and vegetarian status. Statistical significance was set at *p* < 0.05.

## 3. Results

### 3.1. Description of the Study Sample

A total of 176,075 adults completed the HEQ, and met study inclusion criteria, between 2016 and 2022 (Table 1). Respondents were predominantly female (70.4%), Australian residents (62.8%), and aged between 18–24 years (27.7%) or 25–34 years (23.9%). Approximately 13% of respondents followed a vegetarian diet (12.7%). A total of 7087 adults (4.0%) completed the HEQ twice at least three weeks apart and have paired data.

### 3.2. Comparison of Healthy Eating Quiz Scores by Demographic Characteristics

Respondents average ARFS was 33.9/73 (SD = 9.4) (Table 2), which corresponds to the score category 33–38. Linear regression results found that total ARFS was significantly lower in males compared with females (β= −1.89, *p* < 0.001) (Table 3). Total ARFS was significantly higher with increasing age group compared with the 16–17 years age group (e.g., ≥75 years; β = 3.43, *p* < 0.001), in vegetarians compared with non-vegetarians (β = 3.89, *p* < 0.001), and in those who shared main meals with others compared with eating by themselves (e.g., sharing with two or more other people; β = 5.00, *p* < 0.001). Total ARFS was also higher in those living in Australia compared with those living in other countries (β = 3.66, *p* < 0.001), and was higher as socio-economic advantage increased compared with the most disadvantaged (e.g., most advantaged; β = 1.72, *p* < 0.001).

### 3.3. Comparison of Healthy Eating Quiz Scores for Repeat Completers (n = 7087)

The mean ± SD ARFS for repeat completers was 35.3 ± 8.9 on their first completion, and 37.7 ± 9.2 on repeat completion, with a mean change in score of 2.3 ± 6.9 over time (Table 4). Linear regression results found that the change in total ARFS was significantly associated with baseline score category, in both unadjusted and adjusted models (Table 5). In the adjusted model, respondents with a baseline ARFS in the lowest category (<33/73) had the greatest score change (β = 7.04, *p* < 0.001) when compared with those in the highest category (≥47/73).

## 4. Discussion

This study compared diet quality scores of 176,075 adolescents and adults who completed the Healthy Eating Quiz (HEQ) across a six-year period from 2016–2022. Results demonstrated a low mean diet quality score overall; however, diet quality was higher among females, those who share main meals with others, vegetarians, and those living in Australia. Diet quality was also higher with increasing age and socio-economic status. These demographic differences in diet quality are consistent with current evidence [14,17]. Those who completed the HEQ twice also reported an increase in diet quality over time, with the greatest change among those with the poorest baseline diet quality. The findings provide important insight into socio-demographic differences in diet quality, using a large, international database. In particular, the database includes substantial numbers of groups commonly underrepresented in nutrition research, including young adults and males.

The mean diet quality score of individuals in the current study was 34 out of a possible 73, indicating an overall low diet quality in relation to the Australian Dietary Guidelines against which the HEQ was developed. Similarly, prior analysis of preceding HEQ data from 2013–2016 reported a mean diet quality score of 34/73 in a sample of 93,252 who had completed the HEQ in that time [33]. Further, an analysis of national dietary surveys across 187 countries reported overall low diet quality among the adult populations [36]. Collectively, these studies demonstrate that adults globally do not have optimal dietary intakes in comparison with their respective national dietary guidelines or nutrition recommendations.

Socio-demographic differences in diet quality identified in the current study included differences by gender, age, socio-economic status, vegetarian status, country of residence, and how many people individuals consume their main meals with. Consistent with the evidence base [36], females had higher diet quality scores compared with males. Studies have found females to be more health conscious and motivated in regard to a healthy diet than males, which may account for some of this difference [14,15,37]. Diet quality was also found to differ across age groups. Lower diet quality was seen in 18–24- and 25–34-year-olds compared with 16–17-year-olds, mostly contributed by a higher fruit sub-scale score in 16–17-year-olds. This could be influenced by changes such as moving out of home or transitioning from secondary schooling to tertiary education or into the workforce, as found by Winpenny et al. in tracking longitudinal dietary changes across key life transitions of adolescence and young adulthood [26]. Other factors which also influence health behaviours in young adults including changes in finances and new social relationships, can negatively impact dietary intake [26,38]. Diet quality scores were then found to be gradually higher from 35 years onwards, driven by higher vegetable sub-scale scores. This is consistent with evidence from the systematic assessment of national dietary surveys across 187 countries, where 20–29-year-olds were identified as having the least healthy dietary intake compared with older age groups [36].

Disparities in diet quality by socio-economic status were also identified in the current study, with highest diet quality among those of most socio-economic advantage. Previous studies have reported similar findings [21]. For example, an analysis of diet quality in participants of the Observation of Cardiovascular Risk Factors in Luxembourg study found that living below the poverty threshold was associated with consuming a higher energy density diet [39]. Issues of food access and affordability, food and cooking skills, as well as education level, have been identified as moderators of the association between socio-economic status and dietary intake [21,40]. Higher diet quality among those living in Australia compared with living overseas was also found in the current study, mostly driven by higher vegetable sub-scale score. This is not surprising given the HEQ is based on Australian food items and dietary guidelines, which differ to those of other countries. Vegetarians compared with non-vegetarians were found to have a higher diet quality in the current study. Eliminating animal-based foods appears to be associated with greater variety of foods consumed, particularly vegetables and plant-based protein sources, which depending on the scoring, may result in higher diet quality as in the current study [17]. To note though, there are many different types of vegetarian diets [41] and diet quality scores likely differ between these, which the current study was not able to determine. Individuals who consumed main meals with one other person also had higher diet quality compared with those who ate alone, and those who ate with two or more others had higher diet quality again. The social aspect of eating is of major importance, influencing how often, how much and what individuals eat [42]. For example, consuming meals as a family has been linked with healthier dietary intake among adolescents [43], while for older adults social engagement has been identified as a key influence on dietary intake [44].

Change in diet quality over time was assessed for 4% of the sample who chose to complete the HEQ again and whose data were able to be paired. The mean change over time was an increase of 2.3 points, with the change greater amongst those with an initial lower baseline score. That is, those with the lowest diet quality at baseline reported the greatest improvement in diet quality over time. This amount of change is significant when compared with an analysis of diet quality and healthcare usage over 15 years from the Australian Longitudinal Study of Women’s Health (ALWSH) [5]. The ALWSH study found that women made 0.9 fewer health claims and incurred $54 less in health costs for every 1 point increase in diet quality score over time, and this was greater again if the score increase was in the vegetables sub-scale [5]. The brief, automated dietary feedback after completing the HEQ, including score comparison by age and gender and advice on how to increase dietary variety across each sub-scale, may have helped individuals to achieve the positive change in HEQ score over time. This is an important and positive finding, as it suggests that brief, tailored dietary feedback can facilitate improved dietary intake. This idea is supported by evidence from a systematic review of brief, single session, dietary interventions (*n* = 45 studies) in adults which found short-term improvement in fruit, vegetable and fat intakes following intervention, and preliminary evidence of longer-term effectiveness in fruit and vegetable intake and overall diet score [27].

The online, automated nature of the HEQ, including automated dietary feedback and advice, is a major strength of the tool and of the current study, allowing large scale data collection and demonstrating the utility of e&mHealth technology. As a free, online tool, the HEQ and dietary feedback are widely accessible across individuals of varying age, socioeconomic status and geographical location. This is reflected in the diversity of the HEQ database, in particular capturing a high proportion of underrepresented and harder to reach groups in nutrition research such as young adults and males [45,46]. Utilising e&mHealth, the HEQ provides feedback and brief dietary advice, in real-time and that is tailored. As e&mHealth technology advances further [47], the opportunities for online diet index tools such as the HEQ to evolve in terms of access, automation and personalisation will also advance [48], and with it the ability to assess and support dietary behaviour change of individuals and populations.

Additionally, the major strengths include that the HEQ is a validated diet quality assessment tool, and the large size of the dataset collected over six years. The diversity of participants for certain demographic characteristics also adds strength, for example the spread across age groups. For other demographics the sample is not necessarily representative, for example skewed towards females and those with higher socioeconomic status, which is a limitation. Further, as the HEQ is based on Australian Dietary Guidelines which may differ to those of other countries, the scores of non-Australian residents need to be interpreted with this in mind. The large size of the dataset also means that differences appear statistically significant in comparisons of diet quality scores by demographic characteristics which may not be meaningfully different. The ways in which some of the demographic variables are categorised should also be taken into consideration in terms of interpreting the findings. For example, comparing vegetarians versus non-vegetarians does not consider the many variations of dietary patterns within these broad groupings, and comparing Australian versus non-Australian residents does not consider an individual’s country of origin or cultural food influences. In terms of exploring change in diet quality over time, there was a wide variation in the time between HEQ completions and only two completions were considered where some individuals had completed more than twice. Therefore, results should be interpreted with caution.

## 5. Conclusions

Diet quality scores among this large sample of adolescents and adults who completed the online, publicly available Healthy Eating Quiz from 2016–2022 were higher among those with higher socio-economic status, and those who return and complete the quiz again following feedback. Publicly available, brief dietary assessment tools have the potential to improve diet quality at the population level and to contribute important insights into dietary intakes nationally.

## Figures and Tables

**Table 1:** Demographics of adults who completed the Healthy Eating Quiz and met study inclusion criteria from 2016–2022 (*n* = 176,075).

Demographic Characteristic	Total (*n* = 176,075)	Version 3 (*n* = 82,332)	Version 4 (*n* = 93,743)
**Gender**			
Female	124,028 (70.4)	58,590 (71.2)	65,438 (69.8)
Male	51,300 (29.1)	23,742 (28.8)	27,558 (29.4)
Another gender identity	747 (0.4)	0 (0.0)	747 (0.8)
**Age groups**			
16–17 years	19,491 (11.1)	11,719 (14.2)	7772 (8.3)
18–24 years	48,770 (27.7)	23,941 (29.1)	24,829 (26.5)
25–34 years	42,112 (23.9)	19,158 (23.3)	22,954 (24.5)
35–44 years	23,575 (13.4)	9688 (11.8)	13,887 (14.8)
45–54 years	18,593 (10.6)	7768 (9.4)	10,825 (11.6)
55–64 years	14,545 (8.3)	6179 (7.5)	8366 (8.9)
65–74 years	7277 (4.1)	3081 (3.7)	4196 (4.5)
≥75 years	1712 (1.0)	798 (1.0)	914 (1.0)
**Vegetarian**	22,360 (12.7)	9495 (11.5)	12,865 (13.7)
**Number of people main meals are shared with ^a^**			
Only themself	55,502 (32.2)	26,868 (33.4)	28,634 (31.0)
With 1 other person	61,172 (35.4)	27,780 (34.6)	33,392 (36.2)
With 2 or more other people	55,925 (32.4)	25,701 (32.0)	30,224 (32.8)
**Country of residence ^b^**			
Australia	95,407 (62.8)	30,478 (52.4)	64,929 (69.3)
Other	56,484 (37.2)	27,670 (47.6)	28,814 (30.7)
**Index of relative socio-economic advantage and disadvantage (decile) ^c^**			
1–3 (most disadvantaged)	10,569 (14.0)	2857 (14.0)	7712 (14.0)
4–7	27,918 (36.9)	7416 (36.3)	20,502 (37.2)
8–10 (most advantaged)	37,125 (49.1)	10,146 (49.7)	26,979 (48.9)

Values are *n* (%). ^a^ Total *n* = 172,599 due to missing responses. ^b^ Total *n* = 151,891 due to missing responses. ^c^ Total *n* = 75,612 due to participants outside of Australia and missing responses.

**Table 2:** Healthy Eating Quiz total and sub-scale scores by demographic characteristics (*n* = 176,075).

Demographic Characteristic	Total Score/73	Vegetable/21	Fruit/12	Meat/Flesh/7	Plant-Based Protein/6	Grains/13	Dairy/11
**Total**	33.9 ± 9.4	12.3 ± 4.3	5.2 ± 2.8	2.8 ± 1.7	3.0 ± 2.0	5.6 ± 2.3	3.4 ± 2.0
**Gender**							
Female	34.5 ± 9.1	12.5 ± 4.1	5.3 ± 2.7	2.7 ± 1.7	3.1 ± 2.1	5.7 ± 2.3	3.4 ± 2.0
Male	32.6 ± 9.9	11.3 ± 4.5	4.7 ± 2.9	3.0 ± 1.7	2.8 ± 1.8	5.5 ± 2.3	3.5 ± 2.0
Another gender identity	31.5 ± 12.0	10.7 ± 4.9	4.8 ± 3.1	2.0 ± 1.8	3.8 ± 3.0	5.7 ± 2.7	3.0 ± 2.1
**Age groups**							
16–17 years	34.1 ± 10.0	11.1 ± 4.5	6.0 ± 2.9	2.8 ± 1.7	2.6 ± 1.8	6.0 ± 2.3	3.7 ± 2.1
18–24 years	32.4 ± 9.6	11.0 ± 4.4	5.0 ± 2.8	2.4 ± 1.7	2.9 ± 2.1	5.7 ± 2.3	3.3 ± 2.1
25–34 years	33.3 ± 9.6	12.1 ± 4.1	4.7 ± 2.6	2.7 ± 1.7	3.1 ± 2.1	5.7 ± 2.3	3.2 ± 2.0
35–44 years	34.7 ± 9.2	12.9 ± 4.0	5.0 ± 2.7	2.9 ± 1.7	3.1 ± 2.0	5.7 ± 2.4	3.3 ± 2.0
45–54 years	34.9 ± 9.1	13.2 ± 4.0	5.2 ± 2.8	3.1 ± 1.7	3.1 ± 1.9	5.3 ± 2.4	3.4 ± 1.9
55–64 years	35.9 ± 8.9	13.7 ± 3.8	5.5 ± 2.7	3.2 ± 1.7	3.1 ± 1.8	5.2 ± 2.3	3.6 ± 2.0
65–74 years	37.0 ± 8.5	13.9 ± 3.7	6.0 ± 2.6	3.4 ± 1.7	3.2 ± 1.8	5.2 ± 2.1	4.0 ± 1.9
≥75 years	37.5 ± 9.8	13.5 ± 4.2	6.3 ± 2.7	3.6 ± 1.6	3.0 ± 1.7	5.4 ± 2.3	4.3 ± 1.9
**Vegetarian**	37.3 ± 9.3	13.2 ± 4.0	5.6 ± 2.7	0.0 ± 0.0	6.1 ± 3.2	6.2 ± 2.3	2.7 ± 2.1
Non-vegetarian	33.4 ± 9.3	12.0 ± 4.3	5.1 ± 2.8	3.2 ± 1.5	2.6 ± 1.3	5.5 ± 2.3	3.5 ± 2.0
**Number of people main meals are shared with ^a^**							
Only themself	30.9 ± 9.6	10.6 ± 4.5	4.8 ± 2.8	2.3 ± 1.7	3.0 ± 2.1	5.2 ± 2.3	3.2 ± 2.1
With 1 other person	34.8 ± 8.8	12.8 ± 4.0	5.1 ± 2.7	2.9 ± 1.7	3.1 ± 2.1	5.7 ± 2.3	3.4 ± 2.0
With 2 or more other people	35.9 ± 9.0	13.0 ± 4.0	5.6 ± 2.8	3.1 ± 1.6	2.9 ± 1.9	6.0 ± 2.3	3.6 ± 2.0
**Country of residence ^b^**							
Australia	35.1 ± 9.0	13.1 ± 3.9	5.2 ± 2.7	2.9 ± 1.7	3.0 ± 2.2	5.8 ± 2.3	3.4 ± 1.9
Other	32.4 ± 9.6	11.0 ± 4.4	5.1 ± 2.8	2.6 ± 1.7	3.0 ± 1.9	5.5 ± 2.4	3.5 ± 2.1
**Index of relative socio-economic advantage and disadvantage (decile) ^c^**							
1–3 (most disadvantaged)	34.1 ± 9.5	12.8 ± 4.2	5.0 ± 2.9	2.9 ± 1.6	2.8 ± 2.1	5.5 ± 2.3	3.4 ± 1.9
4–7	34.9 ± 9.1	13.1 ± 4.0	5.1 ± 2.8	2.9 ± 1.7	2.9 ± 2.2	5.7 ± 2.3	3.4 ± 1.9
8–10 (most advantaged)	35.8 ± 8.7	13.3 ± 3.8	5.2 ± 2.7	2.9 ± 1.7	3.3 ± 2.2	5.9 ± 2.3	3.4 ± 1.9

Values are mean ± standard deviation. ^a^ Total *n* = 172,599 due to missing responses. ^b^ Total *n* = 151,891 due to missing responses. ^c^ Total *n* = 75,612 due to participants outside of Australia and missing responses.

**Table 3:** Linear regression of Healthy Eating Quiz total scores by demographic characteristics (*n* = 176,075).

Demographic Characteristic	Healthy Eating Quiz Total Score ^a^	
	β-Coefficient	Standard Error
**Gender**		
Reference category = Female		
Male	−1.89 *	0.05
Another gender identity	−2.98 *	0.34
**Age groups**		
Reference category = 16–17 years		
18–24 years	−1.70 *	0.08
25–34 years	−0.81 *	0.08
35–44 years	0.61 *	0.09
45–54 years	0.87 *	0.10
55–64 years	1.83 *	0.10
65–74 years	2.91 *	0.13
≥75 years	3.43 *	0.24
**Vegetarian**		
Reference category = Non-vegetarian		
Vegetarian	3.89 *	0.07
**Number of people main meals are shared with ^b^**		
Reference category = Only themself		
With 1 other person	3.90 *	0.05
With 2 or more other people	5.00 *	0.06
**Country of residence ^c^**		
Reference category = Other		
Australia	3.66 *	0.05
**Index of relative socio-economic advantage and disadvantage (decile) ^d^**		
Reference category = 1–3 (most disadvantaged)		
4–7	0.77 *	0.10
8–10 (most advantaged)	1.72 *	0.10

Values are the results of linear regression analyses. ^a^ No significant difference was found in total ARFS between version 3 and version 4. ^b^ Total *n* = 172,599 due to missing responses. ^c^ Total *n* = 151,891 due to missing responses. ^d^ Total *n* = 75,612 due to participants outside of Australia and missing responses. * *p* value < 0.001.

**Table 4:** Healthy Eating Quiz total and sub-scale scores for repeat completers (*n* = 7087).

	Total Score	Vegetable	Fruit	Meat/Flesh	Plant-Based Protein	Grains	Dairy
First completion	35.3 ± 8.9	13.1 ± 4.0	5.2 ± 2.6	2.8 ± 1.7	3.5 ± 2.5	5.7 ± 2.2	3.5 ± 2.0
Repeat completion	37.7 ± 9.2	13.8 ± 3.9	5.6 ± 2.7	2.9 ± 1.8	3.8 ± 2.6	6.1 ± 2.2	3.7 ± 2.0
Change in score	2.3 ± 6.9	0.8 ± 3.1	0.4 ± 2.2	0.2 ± 1.5	0.3 ± 1.8	0.3 ± 2.0	0.2 ± 1.7

**Table 5:** Linear regression of change in Healthy Eating Quiz total score for repeat completers by baseline score category (*n* = 7087).

Healthy Eating Quiz Category	Change in Healthy Eating Quiz Total Score					
	Unadjusted Model			Adjusted Model ^a^		
	Β-Coefficient	Standard Error	*p*	Β-Coefficient	Standard Error	*p*
Reference category = ≥47						
39–46	2.14	0.29	<0.001	2.16	0.29	<0.001
33–38	4.53	0.29	<0.001	4.60	0.29	<0.001
<33	6.88	0.28	<0.001	7.04	0.29	<0.001

^a^ Adjusted model includes age, gender, and vegetarian status.

## Data Availability

Data used in this study are not publicly available due to rules and restrictions of the approving Ethics Committee.

## Supplementary Materials

The following are available online at https://www.mdpi.com/article/10.3390/nu14194072/s1, Table S1: STROBE-nut: An extension of the STROBE statement for nutritional epidemiology.
